# Yield performance of early-season rice cultivars grown in the late season of double-season crop production under machine-transplanted conditions

**DOI:** 10.1371/journal.pone.0213075

**Published:** 2019-03-20

**Authors:** Jiana Chen, Fangbo Cao, Xiaohong Yin, Min Huang, Yingbin Zou

**Affiliations:** 1 Southern Regional Collaborative Innovation Center for Grain and Oil Crops (CICGO), Hunan Agricultural University, Changsha, P.R. China; 2 Guangxi Key Laboratory of Rice Genetics and Breeding, Rice Research Institute, Guangxi Academy of Agricultural Sciences, Nanning, P.R. China; Shandong University, CHINA

## Abstract

In order to solve the problem of labor shortage in double-season rice production areas, machine transplanting, as opposed to manual transplanting, has become the more popular alternative method in rice cultivation. However, the most existing late rice cultivars are not suitable for machine double-season rice cultivation due to their long duration of growth. Therefore, based on the previous studies we chose early season rice cultivars to meet the needs of machine double-season rice cultivation. In this study, field experiments were conducted during the late season in 2015 and 2016 in Liuyang County, Hunan Province, China. Grain yield and yield-related traits were compared among eight early-season cultivars (Liangyou 6, Lingliangyou 211, Lingliangyou 268, Zhuliangyou 819, Xiangzaoxian 32, Xiangzaoxian 42, Zhongjiazao 17, and Zhongzao 39) in 2015 and four cultivars (Lingliangyou 268, Zhuliangyou 819, Zhongjiazao 17, and Zhongzao 39) in 2016, selected from the highest yielding cultivars grown in 2015. Lingliangyou 268 produced 8–44% higher grain yield than did the other cultivars except Zhongjiazao17 in 2015. This higher grain yield was driven by grain weight and aboveground biomass. The greater aboveground biomass in Lingliangyou 268 was mainly attributed to higher apparent radiation use efficiency (aboveground biomass/incident solar radiation). Our study suggests that improvement in grain weight and apparent radiation use efficiency were critical to the high grain yield of early-season rice cultivars grown in late season under machine transplanting conditions.

## Introduction

Rice is the main staple food of the majority of China’s population. Faced with the continuous reduction of cultivated land area in China and continuous increase in food demand, stabilizing and increasing the planting area of double-season rice and increasing crop yields are important approaches to increase rice production [1]. Soil and climatic conditions in the middle and lower reaches of the Yangtze River are ideal for planting double-season rice and thus is the most important rice production areas in China [2]. Double-season rice cropping is considered an efficient method to improve multiple-crop index and total rice yield [1, 3, 4, 5]. However, the area of double cropping rice has decreased substantially in the last decade in China due to the massive transfer of rural labor [6], the shortage of seasonal labor in double-season rice production areas has become increasingly prominent. Thus, rice production urgently needs the development of planting methods such as machine transplanting to meet the needs of farmers lacking workers [7, 8].

The greatest restriction on the application of machine transplanting of double-season rice is the length of the growing season and the lack of suitable late-season rice cultivars. In machine-transplanted rice production, a high seeding rate is necessary to minimize missing hill rate. The continuous cropping period of late rice coincides with the high temperatures of the growing season. The high temperatures combined with the high seeding rate required by machine transplanting can cause seedlings to grow too fast and too tall which could not match the needs of machine transplanting [7, 9]. Then necessary seedling age for crops to be transplanted by machine is about 15 days, therefore seeds need to be planted 10–15 days later than traditional hand-transplanting and throwing seedlings [10]. However, traditional high-yield late rice cultivars generally mature in 120–130 d and need to be seeded in late June. On the other hand, double-season rice grown under normal climatic conditions need to complete heading by September 15 so that the late-season rice avoids damage from colder temperatures [10, 11]. If we use those traditional late rice cultivars and postpone sowing 10–15 days to shorten the seedling age to meet the requirements of machine transplanting rice, cold damage is more likely to occur at the late stage of rice growth. Therefore, the existing machine transplanting technology cannot be applied in the medium-late maturing high-yield cultivars and most of the existing late rice cultivars are not suitable for machine double-season rice cultivation because of their long growth duration.

Early rice cultivars are more sensitive to temperature than late rice cultivars. In previous studies, we found that the early rice cultivar Zhongjiazao 17 could produce high yields in a 95–102 d growth period when grown in late season under machine transplanting conditions [12]. Thus we selected early rice cultivars to plant in the late season to determine whether they would be suitable for machine double-season rice cultivation. In this study, eight early rice cultivars were grown under machine transplanting conditions in the late season of 2015 and four cultivars (selected from highest yielding cultivars planted in 2015) in the late season of 2016. The objectives of the study were (1) to evaluate the yield performance of early rice cultivars and (2) to identify plant traits associated with high grain yield of early rice cultivars grown under machine transplanting conditions in the late season.

## Materials and methods

### Ethics statements

No specific permissions were required for the activities conducted in this study. The field used in this study is neither privately owned nor protected. The experiments did not involve endangered or protected species.

### Study site

Field experiments were conducted during the late season, from mid-June to late October in 2015 and 2016 in Liuyang County, Hunan Province, China (28°09′N, 113°37′E, 43 m asl). Soil samples from the experimental site were collected from the upper 20-cm layer of the soil in 2015 before beginning the experiments. The soil was clayey in texture and contained 28.69 g organic matter kg^–1^, 2.89 g total N kg^–1^, 24.86 mg available P kg^–1^, and 159 mg available K kg^–1^. The soil pH was 5.95.

### Experiment and measurement

Four hybrid early-season rice cultivars (Liangyou 6, Lingliangyou 211, Lingliangyou 268, and Zhuliangyou 819) and four inbred rice cultivars (Xiangzaoxian 32, Xiangzaoxian 42, Zhongjiazao 17, and Zhongzao 39) were planted in 2015. Of these eight cultivars, the four highest yielding ones were plants in 2016, two were the hybrid early-season rice cultivars (Lingliangyou 268 and Zhuliangyou 819) and two were inbred rice cultivars (Zhongjiazao 17 and Zhongzao 39). Currently, they are widely-grown cultivars in the Yangtze River Valley of China.

The cultivars were arranged in a randomized block design with three replications and a plot size of 30 m^2^. Pre-germinated hybrid seeds and inbred seeds were sown in seedling trays (length × width × height = 58 cm × 25 cm × 2 cm) at respective rates of 110 and 120 g per tray. The seeds were sown on trays on 6 July. Twelve-day-old seedlings were transplanted by a high-speed rice transplanter (PZ80-25, Dongfeng Iseki Agricultural Machinery Co., Ltd., Xiangyang, China) in both years, and transplanting 4–5 seedlings per hill.

Plots received 165 kg N ha^–1^, 100 kg P_2_O_5_ ha^–1^ and 200 kg K_2_O ha^–1^. The total amount of N was applied in three parts: 60% as basal, 30% at mid-tillering and 10% at panicle initiation. Phosphorus was applied as basal fertilizer. Potassium was split equally and applied basally and at panicle initiation. The water regimen was a sequence of shallow irrigation (2–3 cm), midseason drainage (10–15 d) and shallow irrigation. Pests and diseases were carefully controlled using chemicals. Rice sheath blight and false smut were controlled by validamycin. Rice borer, planthopper, and leaf roller were controlled by triazophos, buprofezin, and phoxim, respectively. Because of intensive crop management practices, major pests and diseases stresses that caused yield reductions were not observed in 2015 and 2016. Weeds were controlled by herbicide penoxsulam (Daojie, Dow Agro Sciences, Jiangsu, China) and hand-pulling.

At physiological maturity, ten hills of plants were diagonally sampled in the middle of each plot. Panicle number was counted to calculate panicles per m^2^. Plant samples were separated into straw (including rachis) and panicles. Panicles were hand-threshed. Filled spikelets were then separated from unfilled spikelets by submerging them in tap water. Three subsamples of 30-g filled grain and all unfilled spikelets were manually counted. Dry weights of straw, filled and unfilled spikelets were determined by oven-drying at 70°C to constant weight. Spikelets per panicle, spikelet filling percentage (100 × filled spikelet number/total spikelet number), and the harvest index (filled spikelet weight/aboveground total dry weight) were then calculated. Grain yield was determined from a 5 m^2^ area in each plot and adjusted to the standard moisture content of 0.14 kg H_2_O kg^–1^. Daily grain yield was calculated as the ratio of grain yield to growth duration.

Apparent radiation use efficiency was calculated as the ratio of total aboveground biomass to incident solar radiation during the period from transplanting to maturity. Solar radiation and minimum and maximum temperatures were recorded daily using an on-site automatic weather station (Vantage Pro2, Davis Instruments Corp., Hayward, CA, USA), which was installed about two meters above the level of the field.

### Data analysis

Data were analyzed using analysis of variance, Pearson’s correlation analysis, linear regression analysis, and stepwise regression analysis (Statistix 8.0, Analytical Software, Tallahassee, FL, USA). Significant differences between means were determined by the least significant difference (LSD) test at the 0.05 probability level.

## Results

### Climate conditions

**Fig 1:**
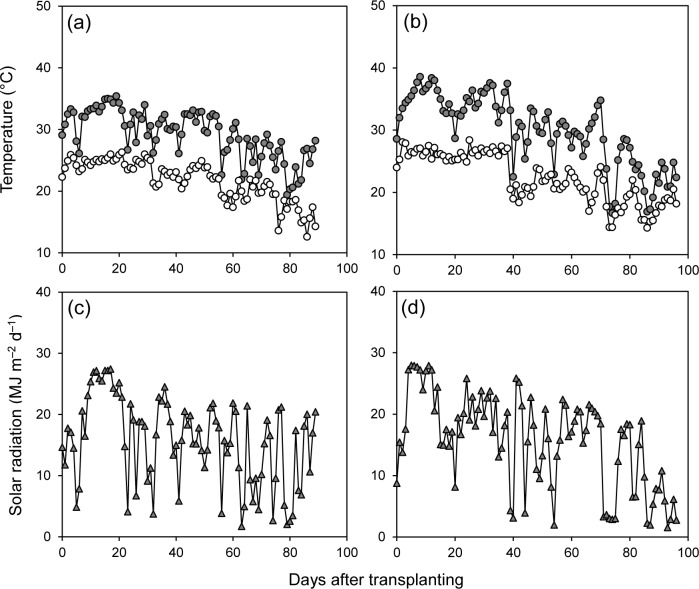
Daily maximum temperature (●), minimum temperature (○) and solar radiation (▲) in 2015 (a, c) and 2016 (b, d).

The seasonal average maximum temperature was 29.7°C in 2015 and 30.5°C in 2016 (Fig 1A and 1B). Seasonal average minimum temperature was 22.3°C in 2015 and 22.6°C in 2016. Seasonal daily solar radiation was 15.9 MJ m^–2^ d^–1^ in 2015 and 16.4 MJ m^–2^ d^–1^ in 2016 (Fig 1C and 1D). The difference in seasonal average daily radiation between the two years was very small.

### Grain yield and yield components

**Fig 2:**
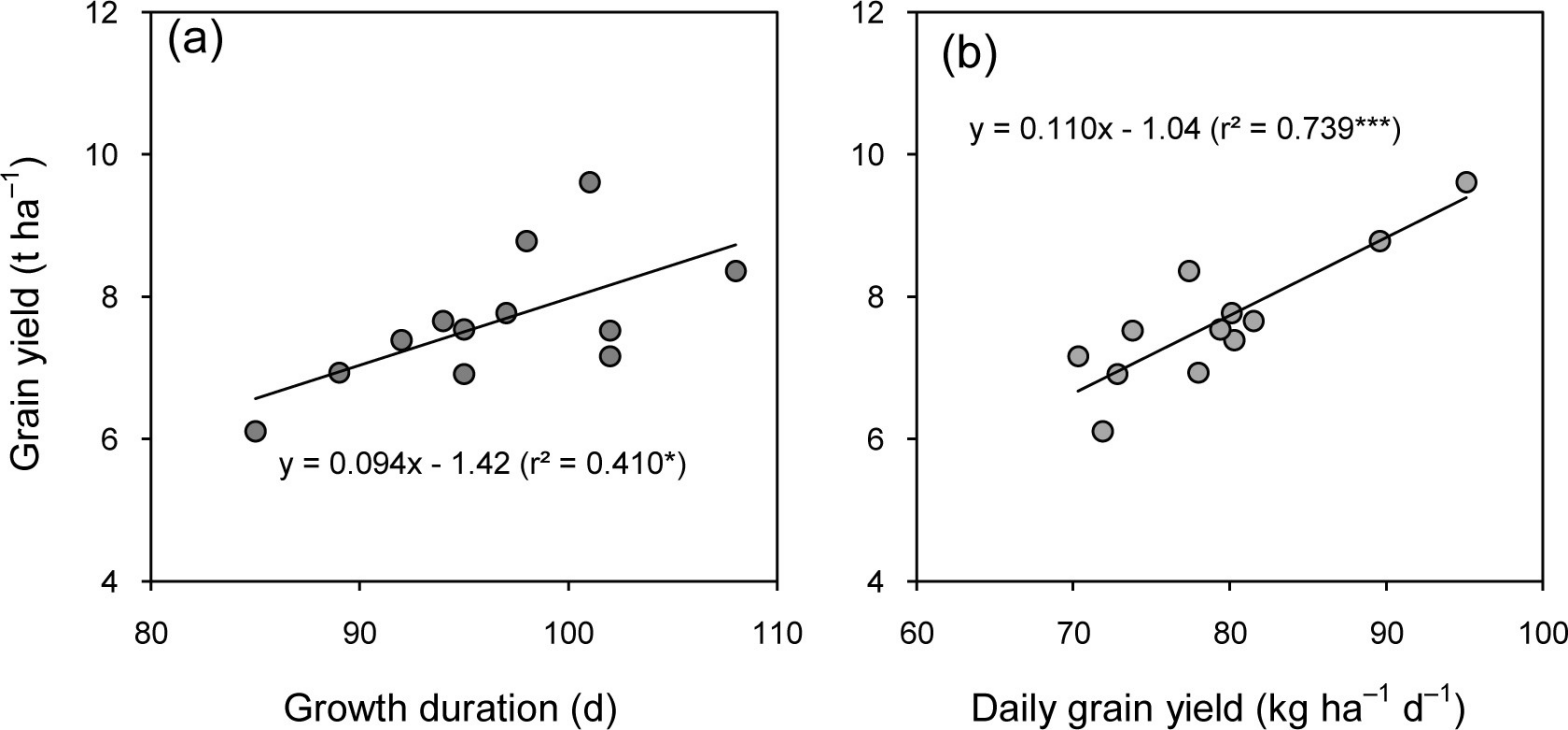
**Relationships of grain yield to growth duration (a) and daily grain yield (b) of early-season rice cultivars grown in the late season under machine-transplanted conditions in 2015 and 2016.** *and *** denote significance at the 0.05 and 0.001 probability levels, respectively.

All the cultivars matured within 108 d (Table 1). Same cultivars grown in both years had different growth durations, Zhongjiazao 17 had the longest growth duration of 101 d in 2015 and Lingliangyou 268 had the longest growth duration of 108 d in 2016. Grain yield significantly differed among cultivars in 2015 and 2016. Zhongjiazao 17 produced the highest grain yield of 9.61 t ha^–1^ in 2015, followed by Lingliangyou 268 which produced the highest grain yield of 8.36 t ha^–1^ in 2016. Furthermore, Lingliangyou 268 produced 17%, 11%, 8% higher yields than Zhongjiazao 17, Zhongzao 39 and Zhuliangyou 819 respectively in 2016. The grain yields of Lingliangyou 268, Zhuliangyou 819, Zhongjiazao 17 and Zhongzao 39 were higher than that of the other four cultivars in 2015, and their differences between the two years were very small except for Zhongjiazao 17. The daily grain yield had nearly the same patterns as yield. Lingliangyou 268 produced daily grain yield of 89.6 kg ha^–1^ d^–1^ in 2015 and 77.4 kg ha^–1^ d^–1^ in 2016, which only significantly lower than Zhongjiazao17 in 2015. There was a strong positive linear correlation between grain yield and growth duration (Fig 2A) and daily grain yield (Fig 2B).

**Table 1:** Growth duration, grain yield and daily grain yield of early-season rice cultivars grown in the late season under machine-transplanted conditions in 2015 and 2016.

Cultivar	Date of physiological maturity (m-d)	Growth duration (d)	Grain yield (t ha^–1^)	Daily grain yield (kg ha^–1^ d^–1^)
2015				
Liangyou 6	10–3	89	6.94 ± 0.12 d	78.0 ± 1.3 cd
Lingliangyou 211	10–6	92	7.39 ± 0.20 cd	80.3 ± 2.2 c
Lingliangyou 268	10–12	98	8.78 ± 0.13 b	89.6 ± 1.3 b
Xiangzaoxian 32	9–29	85	6.11 ± 0.20 e	71.9 ± 2.4 e
Xiangzaoxian 42	10–09	95	6.92 ± 0.18 d	72.8 ± 1.9 de
Zhongjiazao 17	10–15	101	9.61 ± 0.15 a	95.1 ± 1.5 a
Zhongzao 39	10–09	95	7.54 ± 0.11c	79.4 ± 1.1 c
Zhuliangyou 819	10–08	94	7.66 ± 0.03c	81.5 ± 0.3 c
2016				
Lingliangyou 268	10–22	108	8.36 ± 0.02 a	77.4 ± 0.2 ab
Zhongjiazao 17	10–16	102	7.17 ± 0.32 c	70.3 ± 3.1 c
Zhongzao 39	10–16	102	7.53 ± 0.04 bc	73.8 ± 0.4 bc
Zhuliangyou 819	10–11	97	7.77 ± 0.24 b	80.1 ± 2.4 a

Within a column for each year, means followed by the same letters are not significantly different according to LSD (0.05).

The differences in yield components were significant in different cultivars and years (Table 2). The yield components of panicle per m^2^, spikelet filling percentage and grain weight were lower in 2016 than in 2015, but spikelets per panicle was higher in 2016. Lingliangyou 268 had the highest panicle per m^2^ in two years. Zhongjiazao 17 and Zhongzao 39 had the highest spikelets per panicle in 2015 and 2016, respectively. Grain weight of Zhongzao 39 was the highest among the eight cultivars in 2015 but not in 2016, while Lingliangyou 268 had the most consistent highest grain weight of 29.2 mg in 2015 and 29.4 mg in 2016. Grain weight had strong significant positive correlation with grain yield (Table 3). Panicle per m^2^ and spikelet filling percentage had a significant negative correlation with spikelets per panicle.

**Table 2:** Yield components of early-season rice cultivars grown in the late season under machine-transplanted conditions in 2015 and 2016.

Cultivar	Panicles m^–2^	Spikelets panicle^–1^	Spikelet filling (%)	Grain weight (mg)
2015				
Liangyou 6	401 ± 18 c	81 ± 3 de	83.9 ± 0.9 ab	27.9 ± 0.1 c
Lingliangyou 211	505 ± 14 a	71 ± 4 ef	71.5 ± 0.8 c	28.7 ± 0.2 b
Lingliangyou 268	456 ± 6 b	80 ± 2 de	81.1 ± 2.1 b	29.2 ± 0.1 a
Xiangzaoxian 32	445 ± 13 b	64 ± 2 f	88.4 ± 1.9 a	25.8 ± 0.1 e
Xiangzaoxian 42	362 ± 1 d	107 ± 1 b	65.8 ± 1.3 d	26.7 ± 0.0 d
Zhongjiazao 17	402 ± 3 c	127 ± 4 a	75.2 ± 0.6 c	28.8 ± 0.2 b
Zhongzao 39	350 ± 3 d	93 ± 5 c	75.5 ± 2.6 c	29.3 ± 0.2 a
Zhuliangyou 819	452 ± 4 b	82 ± 3 d	73.1 ± 1.3 c	28.7 ± 0.1 b
2016				
Lingliangyou 268	469 ± 20 a	106 ± 5 c	69.3 ± 2.8 a	29.4 ± 0.1 a
Zhongjiazao 17	384 ± 15 bc	119 ± 3 b	70.2 ± 0.6 a	27.8 ± 0.8 b
Zhongzao 39	335 ± 10 c	137 ± 4 a	72.1 ± 1.2 a	27.5 ± 0.2 b
Zhuliangyou 819	427 ± 11 ab	107 ± 0 bc	70.2 ± 2.2 a	27.4 ± 0.1 b

Within a column for each year, means followed by the same letters are not significantly different according to LSD (0.05).

**Table 3:** Correlation coefficients (*r*, *n* = 12) among grain yield and yield components of early-season rice cultivars grown in the late season under machine-transplanted conditions.

Parameter	Grain yield	Panicles m^–2^	Spikelets panicle^–1^	Spikelet filling percentage
**Panicles m**^**–2**^	0.136			
**Spikelets panicle**^**–1**^	0.402	–0.630*		
**Spikelet filling percentage**	–0.210	0.161	–0.582*	
**Grain weight**	0.712**	0.251	0.043	–0.201

*and ** denote significance at the 0.05 and 0.01 probability levels, respectively.

### Biomass production and harvest index

**Fig 3:**
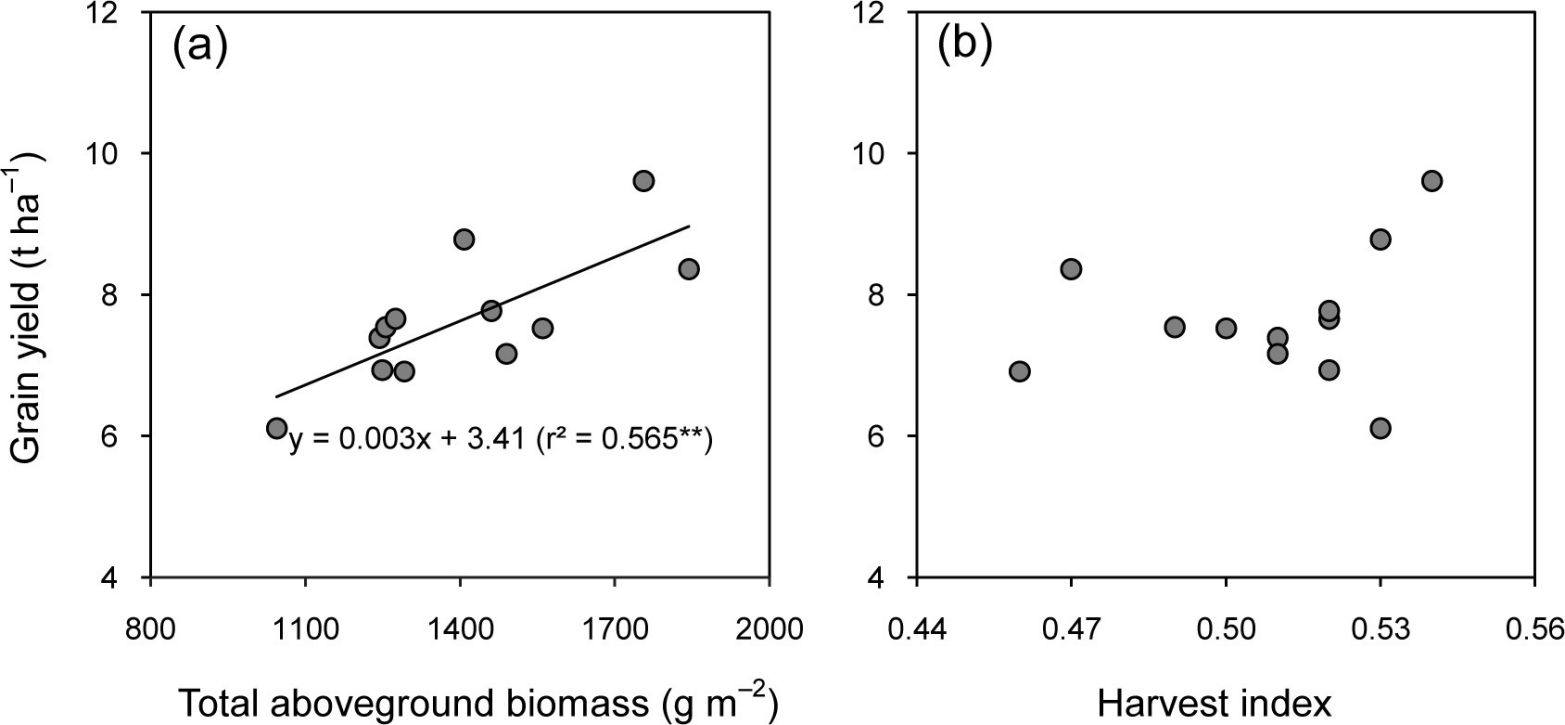
**Relationships of grain yield to total aboveground biomass (a) and harvest index (b) of early-season rice cultivars grown in the late season under machine-transplanted conditions in 2015 and 2016.** ** denote significance at the 0.01 probability levels.

The higher grain yield cultivars also had the higher aboveground biomass (Table 4). Zhongjiazao 17 and Lingliangyou 268 produced the highest aboveground biomass in 2015 and 2016, respectively. Lingliangyou 268 produced 18–26% higher aboveground biomass than the other three cultivars in 2016. Inconsistent differences in harvest index were observed across cultivars and years. There was a strong positive liner correlation between grain yield and aboveground biomass (Fig 3A), while there was no direct correlation between the harvest index and grain yield (Fig 3B).

**Table 4:** Aboveground biomass production and harvest index of early-season rice cultivars grown in the late season under machine-transplanted conditions in 2015 and 2016.

Cultivar	Aboveground biomass (g m^–2^)	Harvest index
2015		
Liangyou 6	1249 ± 19 b	0.52 ± 0.01 ab
Lingliangyou 211	1244 ± 91 b	0.51 ± 0.01 bc
Lingliangyou 268	1407 ± 34 b	0.53 ± 0.01 ab
Xiangzaoxian 32	1044 ± 25 c	0.53 ± 0.01 ab
Xiangzaoxian 42	1291 ± 30 b	0.46 ± 0.00 d
Zhongjiazao 17	1756 ± 56 a	0.54 ± 0.01 a
Zhongzao 39	1255 ± 80 b	0.49 ± 0.01 c
Zhuliangyou 819	1274 ± 52 b	0.52 ± 0.01 ab
2016		
Lingliangyou 268	1843 ± 62 a	0.47 ± 0.00 b
Zhongjiazao 17	1490 ± 47 b	0.51 ± 0.01 a
Zhongzao 39	1560 ± 59 b	0.50 ± 0.00 a
Zhuliangyou 819	1460 ± 29 b	0.52 ± 0.01 a

Within a column for each year, means followed by the same letters are not significantly different according to LSD (0.05).

### Apparent radiation use efficiency

**Fig 4:**
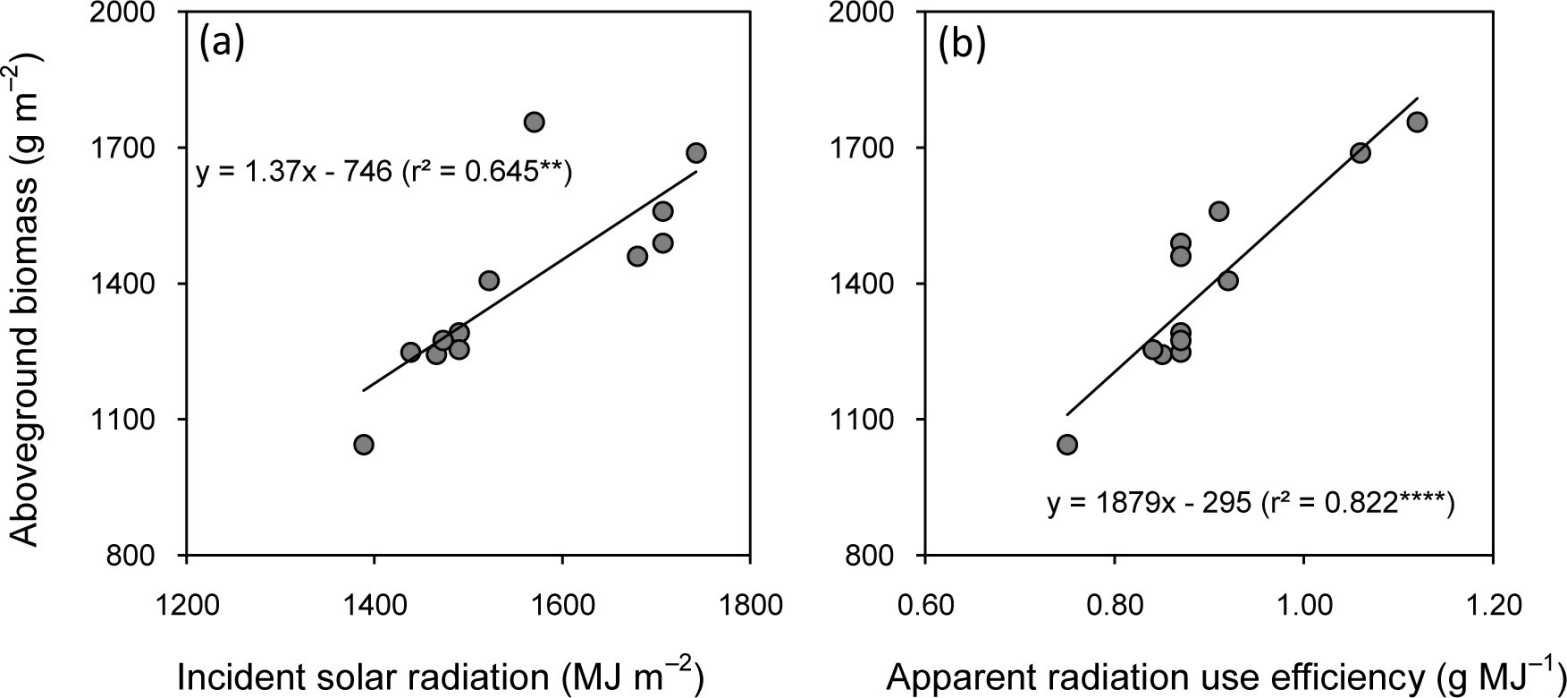
**Relationships of aboveground biomass with incident solar radiation (a) and apparent radiation use efficiency (b) of early-season rice cultivars grown in the late season under machine-transplanted conditions in 2015 and 2016.** **and **** denote significance at the 0.01 and 0.0001 probability levels, respectively.

The apparent radiation use efficiency significantly differed among cultivars and years (Table 5). Zhongjiazao 17 and Lingliangyou 268 had the highest incident solar radiation and apparent radiation use efficiency in 2015 and 2016, respectively. The cultivars with higher aboveground biomass also had the higher incident solar radiation and apparent radiation use efficiencies. There was a strong positive linear correlation of aboveground biomass with incident solar radiation (Fig 4A) and apparent radiation use efficiency (Fig 4B). Apparent radiation use efficiency had more power to explain the variation in aboveground biomass (82%) than incident solar radiation (65%).

**Table 5:** Incident solar radiation and apparent radiation use efficiency of early-season rice cultivars grown in the late season under machine-transplanted conditions in 2015 and 2016.

Cultivar	Incident solar radiation (MJ m^–2^)	Apparent radiation use efficiency (g MJ^–1^)
2015		
Liangyou 6	1438	0.87 ± 0.01 bc
Lingliangyou 211	1466	0.85 ± 0.06 bc
Lingliangyou 268	1522	0.92 ± 0.02 b
Xiangzaoxian 32	1388	0.75 ± 0.02 c
Xiangzaoxian 42	1490	0.87 ± 0.02 bc
Zhongjiazao 17	1570	1.12 ± 0.04 a
Zhongzao 39	1490	0.84 ± 0.05 bc
Zhuliangyou 819	1473	0.87 ± 0.03 bc
2016		
Lingliangyou 268	1742	1.06 ± 0.04 a
Zhongjiazao 17	1707	0.87 ± 0.03 b
Zhongzao 39	1707	0.91 ± 0.03 b
Zhuliangyou 819	1680	0.87 ± 0.02 b

Within a column for each year, means followed by the same letters are not significantly different according to LSD (0.05).

### Stepwise regression

A stepwise regression between grain yield (Y) and the significantly related parameters (X1, grain weight; X2, growth duration; X3, aboveground biomass; X4, incident solar radiation; and X5, apparent radiation use efficiency) showed that the optimal regression equation was Y = –5.7474 + 0.2729*X1 + 6.3628*X5.

## Discussion

Grain yield of early-season rice cultivars grown in the late season under machine transplanting ranged from 6.11 to 9.61 t ha^–1^ with total growth duration of 85 to 108 d across cultivars and years. All the cultivars completed heading before September 15. All tested cultivars can be planted in the late season. However, when taking into account the grain yield, data showed that Lingliangyou 268 produced relative higher grain yield in two years. Analysis of yield components indicates that higher grain weight was responsible for the higher grain yield in Lingliangyou 268. More importantly, the high grain weight was achieved not at the expense of panicle per m^2^ and panicle per number. The importance of grain weight in determining grain yield has been recognized by some researchers [13, 14]. However, some reports show that grain yield is determined by spikelets per m^2^ and/or spikelet number per panicle [10, 15, 16, 17]. The possible reasons for the discrepancies between this study and previous studies are different planting methods and cropping systems. There was a difference in grain yield between years for Zhongjiazao 17, which was lower in 2016 than in 2015. The lower grain yield of Zhongjiazao 17 in 2016 was mainly attributed to lower yield components and biomass production than in 2015. The difference in yield was probably due to the weak compensation mechanism of Zhongjiazao 17 [18].

In our study, the growth duration of Lingliangyou 268 was only 98 d and 108 d in 2015 and 2016, respectively, which are shorter growth periods than the traditional late-rice cultivars generally that mature in 120–130 d [11]. Studies have shown that short-duration rice cultivars grow rapidly to produce more biomass [10, 19]. Compared with the harvest index, total aboveground biomass was more important in explaining the higher yield in Lingliangyou 268, and there was a strong positive linear correlation between grain yield and aboveground biomass, the results are consistent with previous studies [4, 5, 20, 21, 22], which indicated that the improvement in rice yield might be driven by higher biomass production. The growth duration of early season cultivars grown under machine transplanting in the late season can vary because plant growth depends on temperature [12]. In this study, we observed a 1–10 d difference in growth duration of cultivars between the two years. The high biomass production in 2016 was primarily attributed to growth duration.

Generally, high grain yield is attributed to high incident solar radiation [23, 24] and is supported by our results. We also concluded that higher increased incident solar radiation and apparent radiation use efficiency were critical factors to increasing aboveground biomass, which results in higher yield. Radiation use efficiency is a more commonly used indicator than apparent radiation use efficiency, which related to intercepted solar radiation by the canopy and intercepted percent. However, some researchers have reported that high biomass production is mainly caused by intercepted solar radiation rather than radiation use efficiency, the radiation intercepted is more closely related to incident radiation than to intercepted percent [24, 25]. Qin [26] reported that the intercepted percent for late season rice was 66%–83%. Our calculated average radiation use efficiency was 1.08–1.36 g MJ^–1^ based on Qin’s results, the number was very close to the radiation use efficiency values which estimated in previous studies [27, 28]. Thus, apparent radiation use efficiency is feasible and an easier measure to use to reflect the use efficiency of radiation. However, in our study the crop’s radiation use efficiency was not measured, thus the relationship of the apparent radiation use efficiency and radiation use efficiency needs to be further studied.

## Conclusion

Among the tested early-season rice cultivars, Lingliangyou 268 is better to adapt to machine transplanting in the late season. Selecting early-season rice cultivars grown under machine transplanting in the late season, they should meet the following requirements: (1) have high grain weight and (2) high apparent radiation use efficiency.
