# Correction: Yield performance of early-season rice cultivars grown in the late season of double-season crop production under machine-transplanted conditions

**DOI:** 10.1371/journal.pone.0269289

**Published:** 2022-05-26

**Authors:** Jiana Chen, Fangbo Cao, Xiaohong Yin, Min Huang, Yingbin Zou

After the publication of this article [1], the *PLOS ONE* journal was notified about the omission of the minimal dataset. The missing information can be viewed below. The data has been assessed by senior members of the journal staff and a member of the *PLOS ONE* Editorial Board. With this correction, the relevant data are now provided.

## Supporting information

S1 File Supplementary dataset. This file includes the minimal dataset underlying Tables 1, 2, 4, and 5.
(XLSX)
